# Complete Genome Sequence of the Corallopyronin A-Producing Myxobacterium Corallococcus coralloides B035

**DOI:** 10.1128/MRA.00050-19

**Published:** 2019-04-25

**Authors:** Sarah Bouhired, Oliver Rupp, Jochen Blom, Till F. Schäberle, Andrea Schiefer, Stefan Kehraus, Kenneth Pfarr, Alexander Goesmann, Achim Hoerauf, Gabriele König

**Affiliations:** a Institute for Pharmaceutical Biology, University of Bonn, Bonn, Germany; b Institute for Medical Microbiology, Immunology and Parasitology, University Hospital Bonn, Bonn, Germany; c Bioinformatics and Systems Biology, Justus-Liebig University Giessen, Giessen, Germany; d Institute for Insect Biotechnology, Justus-Liebig University Giessen, Giessen, Germany; e German Center for Infection Research (DZIF), Partner Site Bonn-Cologne, Bonn, Germany; f Department of Bioresources, Fraunhofer Institute for Molecular Biology and Applied Ecology, Giessen, Germany; University of Arizona

## Abstract

Myxobacteria are a source of unique metabolites, including corallopyronin A (CorA), a promising antibiotic agent in preclinical development for the treatment of filariasis. To investigate the production of CorA on the genetic level, we present the complete 9.6-Mb genome sequence of the CorA producer Corallococcus coralloides B035.

## ANNOUNCEMENT

Myxobacteria are fascinating Gram-negative *Deltaproteobacteria* with remarkable features that include social behavior, gliding on solid surfaces, and formation of fruiting bodies under starvation conditions (1, 2). Most noticeable are the tremendous biosynthetic capabilities of these microorganisms, making them an important source for novel biologically active metabolites (3). The therapeutic spectrum of myxobacterial natural products includes antibacterial, antifungal, antiplasmodial, and antitumor activities (4). A promising myxobacterial compound is the antibiotic agent corallopyronin A (CorA), isolated in 1985 from Corallococcus coralloides Cc c127 (5). CorA is a specific inhibitor of the bacterial DNA-dependent RNA polymerase (RNAP), with a new mode of action compared to rifampin and with efficacy against obligate intracellular Gram-negative *Wolbachia* endosymbionts found in many filarial nematodes that infect humans, causing lymphatic filariasis and river blindness. Additionally, CorA is also active against Gram-positive bacteria, including methicillin-resistant Staphylococcus aureus (MRSA) and rifampin-resistant S. aureus (5–7).

*C. coralloides* DSM 2259, sequenced in 2012, was the first member sequenced from the genus *Corallococcus*, and its genome is the only complete one available for this genus (8). However, this strain does not carry the biosynthetic gene cluster (BGC) corresponding to CorA biosynthesis. The biosynthetic genes (*corA* through *corO*) responsible for the formation of CorA were identified in strain *C. coralloides* B035 by using Sanger sequencing of three cosmids (9).

For a better understanding of the CorA producer, we sequenced and annotated the entire genome of *C. coralloides* B035, which was isolated by our group from a Belgian soil sample (9). We cultured *C. coralloides* B035 in MD1 medium+glucose until the onset of fruiting body formation (10). Subsequently, genomic DNA was isolated from the collected and homogenized cells. The isolation procedure described by Kohler et al. was used in order to achieve large amounts of high-molecular-weight genomic DNA (>40 kb) required for PacBio single-molecule real-time (SMRT) sequencing (11). A shotgun library of the genomic DNA was prepared and transferred to two SMRT cells prior to *de novo* sequencing with the PacBio RS II platform at Eurofins Genomics using P6 chemistry.

The sequence reads (filtered subreads, 282,028; 350-fold coverage; average read length, 11,670 bp) were assembled following the HGAP workflow (version 4, with pbsmrtpipe version 0.44.8), including preassembly, assembly, and consensus polishing (default parameters were used for all software, and the GenomeLength parameter was set to 16 Mb to increase the assembly coverage). This resulted in a single scaffold of contiguous DNA with 9,587,888 bp and a GC content of 70%. Genome annotation was performed with Prokka software (version 1.12), allowing functional assignment of 63% of the genes in the entire genome, whereas 37% of the genes were assigned to be hypothetical proteins. This yielded 7,624 protein-coding genes, 63 tRNA genes, and 9 rRNA operons for the genome of *C. coralloides* B035.

Size and genetic content are in the ranges of those for other completely sequenced myxobacterial genomes, with genome sizes between 8.9 and 14.7 Mb (12, 13). The *C. coralloides* B035 genome most closely matches that of *C. coralloides* DSM 2559 (10.0 Mb), with an average nucleotide identity of 96.6% (calculated on the EDGAR platform) (14). Investigations of the biosynthetic potential with antiSMASH (version 4.3.0) predicted 81 BGCs for B035 and 84 BGCs for DSM 2259 (15). In contrast to the genome of DSM 2259, the genome of B035 harbors BGCs for indole and siderophore biosynthesis in addition to a *trans*-acyltransferase (*trans*-AT) polyketide synthase (PKS) gene cluster. Further investigations of the BGC encoding a *trans*-AT PKS revealed that the genes are identical to those in the previously identified CorA gene cluster (*corA* through *corO*) and are responsible for CorA formation (9).

### Data availability.

The complete genome sequence has been deposited at DDBJ/ENA/GenBank under the accession number CP034669. Raw sequencing data have been deposited under BioProject accession number PRJNA509558.
